# Numerical Investigation of MWCNT Effects on Elastic Properties of PA6/POM Blends

**DOI:** 10.3390/polym18050644

**Published:** 2026-03-06

**Authors:** Katarina Pisačić, Srečko Glodež, Aleš Belšak

**Affiliations:** 1 Faculty of Mechanical Engineering, University of Maribor, 2000 Maribor, Slovenia; katarina.pisacic@unin.hr (K.P.); srecko.glodez@um.si (S.G.); 2 Department of Mechanical Engineering, University North, 48000 Koprivnica, Croatia

**Keywords:** hybrid polymer, POM, PA6, representative volume element, numerical analysis

## Abstract

To ensure the viability of polymer materials, given the properties and limitations of polymers, hybrid materials have been developed that blend the features of all included components. Researchers have not explored the impacts of the length aspect ratio of nanofillers on the mechanical properties of hybrids in great detail previously. Multi-walled carbon nanotubes are a valuable option because they exhibit improved mechanical properties. Using numerical simulation, the impacts of nanofiller content and the size aspect ratio on two base materials—polyamide 6, polyoxymethylene—and their blends, were determined as a function of the volume ratio, the MWCNTs aspect ratio and the base material blend composition. Numerical analysis employed the ANSYS Material Designer. Random samples of chopped-fibre representative volume elements were generated, meshed and analysed by finite element analysis to obtain the Young’s modulus and Poisson’s ratio for each sample. The results showed a generally linear dependence. Rises in both aspect ratio and volume fraction of MWCNTs increased the Young’s modulus up to 46% and decreased the Poisson’s ratio up to 1.6%. The findings suggest that although the impact of the aspect ratio is not as large as that of the volume ratio, longer MWCNTs are preferable.

## 1. Introduction

Polymer gears are a viable industrial solution. Their positive characteristics include vibration damping, low mass, low noise and chemical resistance, but they lack mechanical strength and wear resistance compared to steel.

Polyamide 6 (PA6) is an engineering plastic renowned for its favourable mechanical and thermal properties, rendering it suitable for a wide range of applications. PA6 characteristics can be influenced by temperature, the strain rate and the incorporation of additives or copolymers. PA6 exhibits high tensile strength and stiffness, both of which can be enhanced by reinforcement with materials such as carbon nanotubes (CNTs) and graphene [1]. The material exhibits improved toughness when modified by the addition of boron-containing compounds, which significantly improves its impact strength [2]. The PA6 melting temperature (Tm) can be adjusted via copolymerization. The introduction of aromatic imide structures increased the glass transition temperature (Tg) and improved thermal stability [3]. The thermomechanical behaviour of PA6 was highly dependent on the strain rate and temperature, with softening observed at high strain rates because of heat generation [4]. The addition of polyetheramine enhanced the melt flowability of PA6 without compromising the mechanical strength, thus facilitating easier processing [5]. Although PA6 has mechanical and thermal properties appropriate for industrial applications, flammability remains a concern, necessitating ongoing research on flame-retardant modifications that would broaden the application scope.

Polyoxymethylene (POM) is an engineering thermoplastic that exhibits thermal stability and chemical resistance. The structure contributes to its high crystallinity and low friction, making POM suitable particularly in the automotive and other industries. POM exhibits high tensile and flexural moduli (which can increase significantly with the incorporation of natural fibres, enhancing mechanical performance [6]), evidences higher toughness and fracture resistance (which can be optimised by adjusting the molecular weight during processing [7]), and has a melting point of approximately 162 °C and a decomposition temperature of approximately 266 °C, indicating suitability for high-temperature applications [8]. The material behaves as a non-Newtonian fluid, exhibiting shear-thinning behaviour, which is advantageous during processing [8]. POM is resistant to a wide range of solvents, chemicals and fuels at room temperature, rendering it a reliable alternative to metals for various applications [9]. Although these properties of POM make it useful for many applications, the need for formaldehyde treatment during production raises environmental concerns. This aspect necessitates ongoing research on more sustainable alternatives and processing methods.

Hybridisation of polyamide (PA) with POM has garnered increasing attention because this may enhance the mechanical and thermal properties of POM. Such combinations leverage the strengths of both polymers, improving the performance characteristics. The addition of PA to POM significantly enhances the mechanical properties of POM, such as the notched impact strength and elongation at break, particularly when compatibilisers such as maleic anhydride-grafted ABS are used [10]. The nucleation effect mediated by PA reduces spherulite size in POM, improving crystallisation rates and the overall mechanical stability [11]. The rheological properties of POM-PA blends differ from those of the individual polymers and are influenced by the mixing ratio and method. This can affect both processing and application performance [12]. Morphologically, POM-PA blends exhibit more uniform elastomer particles and increased amorphous regions, contributing to enhanced toughness and flexibility [13]. Although hybridisation of PA with POM offers numerous advantages, challenges such as compatibility and the processing conditions must be addressed to exploit fully the potential of hybridisation.

The use of nanoparticles as fillers for various polymer hybrid materials has been described in the literature. In the work [14], nanoparticles were used to enhance the toughening of epoxy resin. In [15], the effects of nanotube waviness on composite stiffness were studied. A finite element model was used to investigate the volume fraction and waviness impacts, and the results show that waviness decreased the longitudinal Young’s modulus compared to that of straight nanotubes. The fibre curvature minimally affected the Poisson’s ratio, whereas the fibre volume fraction influenced both the longitudinal Young’s modulus and the Poisson’s ratio.

A multi-stage hierarchical micromechanical approach was used to predict the elastic properties of a composite. The authors [16] explore the effects of carbon fibre (CF) and CNTs characteristics on the mechanical properties of such materials. The addition of CNTs increased the transverse elastic modulus without affecting the longitudinal properties.

In [17], the mechanical properties of nanocomposites containing multi-walled carbon nanotubes (MWCNTs) were investigated. Finite element analysis (FEA) was used to assess the effects of various parameters. The study compared MWCNTs and single-walled carbon nanotubes (SWCNTs) in terms of reinforcement efficiency. The results agreed with the experimental data for the Young’s modulus predictions.

The authors of [18] investigated the effects of the CNT weight fraction on nanocomposite fracture energy. To this end, numerical models with 0.1, 0.2 and 0.5 wt% were employed. The fracture energy was determined using finite element simulations and micromechanics. Mixed-mode loading was applied to determine the crack growth paths, and the numerical results were compared with experimental measurements reported in the literature.

One study [19] investigated the effects of CNT volume fraction and dispersion on nanocomposites using finite element methods to simulate six loading conditions. A dispersion quantification technique was employed to assess nanotube distribution, and the results showed significant dispersion effects at volume fractions above 5%.

CNT bundles of nanocomposites are lightweight, flexible, and stiff. One study [20] investigated the effects of straight and rope-shaped bundles on mechanical properties. FEA models were used to analyse the dimensions and reinforcing efficiency of CNTs in various matrices, and the results indicated that an increased Young’s modulus influenced the stiffening of both longitudinal and transverse fibres.

One study [21] investigated free vibration and stress wave propagation in two types of nanotubes. The results showed that the stiffness trends differed for short wavy CNTs and long straight CNTs and that X-CNTRC cylinders exhibited the highest frequency parameters under specific conditions.

In [22], a semi-continuum model was used to efficiently predict the behaviour of graphene-reinforced polymer nanocomposites. Graphene nanoplatelets (GNPs) and CNTs enhanced the mechanical properties of polymer nanocomposites, and the new micromechanical model presented in [23] effectively predicted the elastic properties. This study revealed the importance of nano-filler dispersion and explored the synergistic effects of GNPs and CNTs.

In [24], multiple simulation scales that aided the efficient design of polymer−clay nanocomposites (PCNs) were integrated, and in [25], the effect of graphene oxide on the mechanical properties of CNT nanocomposites was investigated. A further study [26] shows a model of the tensile behaviour of polymer-grafted nanoparticle networks.

In this study, PA6/POM blends with added MWCNTs are analysed using FEA in ANSYS 2019 R1 Material Designer. Using the representative volume element (RVE) and the chopped fibre model, the elastic properties of POM, PA6 and their blends with varying MWCNT volume fractions are determined.

## 2. Materials and Methods

Various groups [27,28,29,30,31,32,33,34] adopted different approaches to enhance the properties of polyamide-based materials by adding carbon fibres. The filler content was sometimes up to 40% of the volume ratio. The authors of [35,36,37,38,39,40,41,42,43,44,45,46,47,48,49,50,51] used nanoparticles, nano-fillers and micro-fillers as additives to PA6.

The advantages of polymer hybrids include enhanced performance, improved specific properties (Young’s modulus, mechanical strength, etc.) and attainment of a desired combination of characteristics using two or more polymers at a reduced cost. The goal of this process is to simplify the formulation and increase productivity. A miscible blend of two polymers exhibits properties intermediate between those of the two unblended polymers. Most polymer blends tend to be immiscible; to address this issue, compatibilisers are typically added to enhance the interfacial adhesion between the polymers [52]. Various fillers have also been included to further improve the properties of hybrid polymers, like mechanical strength and Young’s modulus. Polyoxymethylene hybrids with nanofillers are described in articles [52,53,54,55,56,57].

Given the various possible combinations of filler materials, it is not easy to select a specific filler material. Table 1 shows the decision matrix used to select the most appropriate nanofiller type. The know-how derived from a number of recent numerical simulations emphasises that this topic is currently novel and that there have been continuous advances in the application of nanomaterials.

CNTs were chosen as the most appropriate filler type to enhance the base material properties of the matrix polymer material. After several attempts to use SWCNTs, the meshing procedure could not be performed by either the Material Designer or the Static Structural module of ANSYS. The shape of SWCNTs dictates that the diameter is very small and the aspect ratio (i.e., the ratio of fibre diameter to length) is very large (approximately 1000). Such models cannot be solved by ANSYS Material Designer because of irregularities in the mesh and overly small dimensions that enter the pico-range. MWCNTs with larger diameters and smaller aspect ratios were therefore chosen to overcome the issues with large aspect ratios.

After choosing the MWCNTs, the geometrical properties and volume ratio were next selected. For this task, FEA (in the form of a numerical experiment) was used. In terms of the material input data, the mechanical properties of the blend were calculated using the rule of mixtures. A numerical experiment was performed with the filler material added to the base polymer at different volume ratios. The initial ratios were determined using the published data cited above. To determine the optimal composition, the average modulus of elasticity was calculated for a representative volume element (RVE) [58].

The material properties used for the numerical experiment were obtained from various sources, as a variety of trade-marked materials are available with different mechanical properties. The main sources of the data were internet vendors and the literature [59,60,61,62,63,64,65,66].

For PA6/POM with a 50:50 mass ratio, volume fractions were calculated, as were the properties of the blended material. We used the law of mixtures—the Voigt model of the rule of mixtures [67], assuming that the load was shared equally among the components and that both components in the blend experienced the same strain. To obtain the real properties of the blend, experiments will be conducted later. The values were calculated using Mathcad as follows:

The input values were as follows:

(1) $$\begin{array}{cc} E_{\mathrm{PA6}}=1.6\;GPa, & E_{\mathrm{POM}}=2.66\;GPa,\\ \rho_{\mathrm{PA6}}=1.14\;\frac{g}{{\mathrm{cm}}^{3}}, & \;\rho_{\mathrm{POM}}=1.41\;\frac{g}{{\mathrm{cm}}^{3}}\\ \upsilon_{\mathrm{PA6}}=0.35, & \;\upsilon_{\mathrm{POM}}=0.39\\ f_{\mathrm{PA6}}=0.5, & \;f_{\mathrm{POM}}=0.5 \end{array}$$

Formulas (2)–(6) show the calculations of the blend properties, where the following symbols are used: modulus of elasticity (*E*), density (*ρ*), weight fraction (*f*) and volume fraction (*V*).

(2) $$V_{\mathrm{POM}}=\frac{f_{\mathrm{POM}}}{f_{\mathrm{POM}}+\left(\frac{\rho_{\mathrm{POM}}}{\rho_{\mathrm{PA6}}}\right)\cdot \left(1-f_{\mathrm{POM}}\right)}=0.447$$

(3) $$V_{\mathrm{PA6}}=\frac{f_{\mathrm{PA6}}}{f_{\mathrm{PA6}}+\left(\frac{\rho_{\mathrm{PA6}}}{\rho_{\mathrm{POM}}}\right)\cdot \left(1-f_{\mathrm{PA6}}\right)}=0.553$$

(4) $$\nu_{\mathrm{blend}}=V_{\mathrm{POM}}\cdot \nu_{\mathrm{POM}}+V_{\mathrm{PA6}}\cdot \nu_{\mathrm{PA6}}=0.368$$

(5) $$E_{\mathrm{blend}}=V_{\mathrm{POM}}\cdot E_{\mathrm{POM}}+V_{\mathrm{PA6}}\cdot E_{\mathrm{PA6}}=2.074\;\mathrm{GPa}$$

(6) $$\rho_{\mathrm{blend}}=V_{\mathrm{POM}}\cdot \rho_{\mathrm{POM}}+V_{\mathrm{PA6}}\cdot \rho_{\mathrm{PA6}}=1.261\frac{g}{{\mathrm{cm}}^{3}}$$

Although the literature gives a wide range of mechanical properties for each material, the material properties used for the engineering data in this FEA are limited to a single range, as listed in Table 2.

**Figure 1:**
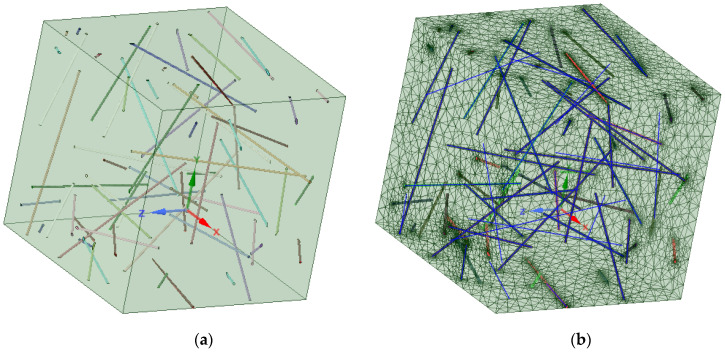
RVE element of a 0.2% volume fraction of MWCNTs: (**a**) geometry of RVE; (**b**) meshed RVE.

The MWCNTs’ diameter was set to 50 nm, and the aspect ratio was set to 100 [68] for the RVE, as shown in Figure 1. Figure 1 shows one randomly generated set. A random generator can be seeded to enable easier control of the FEA. The Material Designer (MD) interface offers limited possibilities for meshing; in this case, the conformal and periodic mesh options were selected with the maximum mesh size of 1 μm. The size of the RVE was not given; the RVE was generated automatically for each design point, the cube sepresents a fraction of material where colored lines represent MWCNTs.

The numerical experiment employed a full factorial design with three factors at three levels. In this experimental setup, every possible combination of factor levels was tested. The sample geometry was designed using a chopped fibre option and random geometry. It is important to note that the MD was set to use 10 different samples per design point.

**Figure 2:**
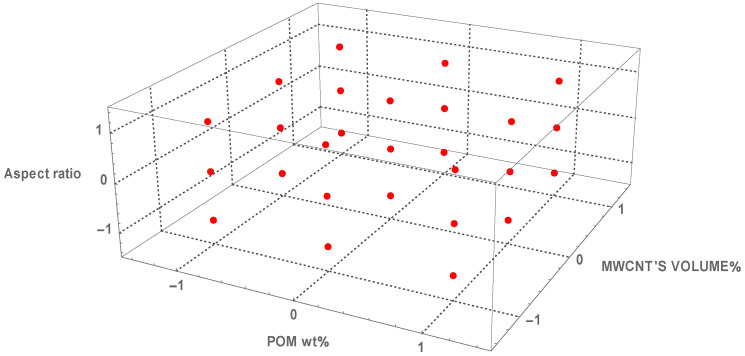
Visualisation of the three-factor full factorial design of the experiment.

The experimental samples were designed to accommodate different volume fractions for three particle aspect ratios. The factors were as follows: the POM weight ratio in PA6, the volume fraction of MWCNTs in the base material, and the MWCNT aspect ratio. This design explores all interactions and main effects and affords a high predictive accuracy. The full factorial design cube is shown in Figure 2. The axes represent input factors and red dots represent design points.

Aspect ratios were chosen based on the availability of MWCNTs on vendors’ websites and the experimental design, the limitations of Material Designer meshing capabilities were also considered.

Table 3 shows the composition of each sample of this numerical experiment. A relatively low volume fraction was chosen because of the mixing issues that arise when using a large fraction of CNTs. The first part of the designations stands for the composition of the base material PA6, POM or their 50:50 blend, the second part is a volume fraction of MWCNTs (shortened to nanotubes or NTs), and the third part is for the aspect ratio.

## 3. Results

The MD of ANSYS 2019 has only limited options, and the results of an anisotropic material analysis come in the form of a stiffness matrix from which the values of the elasticity modulus and Poisson’s ratio can be calculated in all three directions. The values calculated by ANSYS lie on the diagonal and below, whereas values above the diagonal are transposed below the diagonal. The results and the values for different fibre volume fractions are given in Table 4. The results for the volume fractions of 0.5%, 0.1% and 0.16% are given in columns 2, 4 and 6, respectively. The additional results in columns 3, 5 and 7 are generated automatically and are not considered further.

The sample calculations using Formulas (7) and (8) and the results of Formula (9) show the stiffness matrix D and the process used when calculating the compliance matrix S, the Poisson’s ratio and Young’s modulus for the sample PA6/05NT1.

(7) $$D=\left(\begin{array}{cccccc} 2892 & 1501.6 & 1512.8 & 7.7168 & 16.709 & 26.601\\ 1501.6 & 3004.5 & 1506.3 & 10.665 & 23.126 & 3.7436\\ 1512.8 & 1506.3 & 2974.7 & 10.001 & 43.252 & 35.532\\ 7.7168 & 10.665 & 10.001 & 709.15 & 3.0886 & 16.533\\ 16.709 & 23.126 & 43.252 & 3.0886 & 714.7 & 11.909\\ 26.601 & 3.7436 & 35.532 & 16.533 & 11.909 & 720.36 \end{array}\right)$$

(8) $$S\;:=D^{-1}=\left(\begin{array}{cccccc} 0.0005316 & -0.0001745 & -0.000182 & -0.0000004 & 0.0000044 & -0.0000083\\ -0.0001745 & 0.0005034 & -0.0001663 & -0.0000036 & -0.0000023 & 0.0000117\\ -0.000182 & -0.0001663 & 0.0005134 & -0.0000023 & -0.0000211 & -0.0000178\\ -0.0000004 & -0.0000036 & -0.0000023 & 0.001411 & -0.0000053 & -0.0000322\\ 0.0000044 & -0.0000023 & -0.0000211 & -0.0000053 & 0.001408 & -0.0000221\\ -0.0000083 & 0.0000117 & -0.0000178 & -0.0000322 & -0.0000221 & 0.0013904 \end{array}\right)$$

(9) $$\begin{array}{ccc} E_{1}=\frac{1}{S_{1,1}}=1881.082\;\;\mathrm{MPa} & E_{2}=\frac{1}{S_{2,2}}=1986.43\;\mathrm{MPa} & E_{3}=\frac{1}{S_{1,1}}=1947.689\;\mathrm{MPa}\\ G_{1}=\frac{1}{S_{4,4}}=708.716\;\mathrm{MPa} & G_{2}=\frac{1}{S_{5,5}}=713.861\;\mathrm{MPa} & G_{3}=\frac{1}{S_{6,6}}=719.216\;\mathrm{MPa}\\ \upsilon_{1}=\frac{1}{S_{4,4}}=0.328 & \upsilon_{2}=\frac{1}{S_{5,5}}=0.342 & \upsilon_{3}=\frac{1}{S_{6,6}}=0.33 \end{array}$$

The results reveal anisotropic material properties. To represent each result set as a singular equivalent value, the equivalent values were calculated as the average value, using Formulas (10) and (11):

(10) $$E_{\mathrm{average}}=\frac{E_{1}+E_{2}+E_{3}}{3}=1938.4\;\mathrm{MPa}$$

(11) $$\nu_{\mathrm{average}}=\frac{\nu_{12}+\nu_{13}+\nu_{23}}{3}=0.3336$$

After calculating the mean Poisson’s ratio and Young’s modulus for each set of results, Table 5 was compiled, where the first column shows the sample aspect ratios. The average values of the Young’s modulus are given in columns 2, 3 and 4. Column 2 shows the results for samples with a 0.005 volume ratio of MWCNTs, column 3 shows the results for samples with a 0.01 volume ratio of MWCNTs, and column 4 shows the results for samples with a 0.016 volume ratio of MWCNTs. Values of the Poisson’s ratio are given in columns 5, 6 and 7. Column 8 shows the base material compositions.

**Figure 3:**
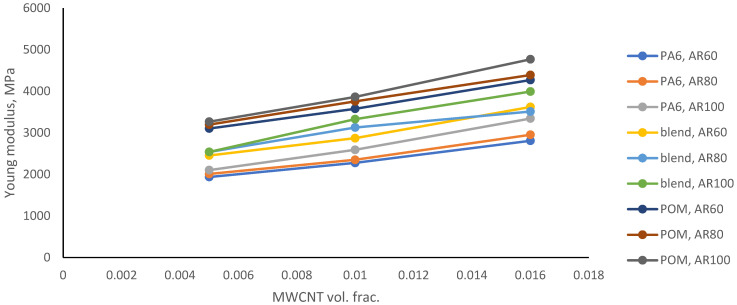
Average Young’s modulus for various AR and base material blends.

**Figure 4:**
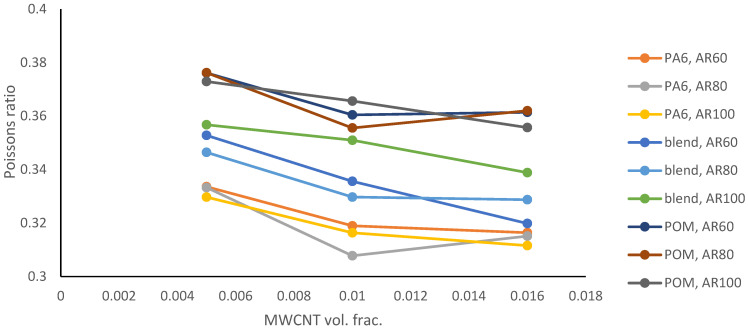
Average Poisson’s ratio for various base materials and aspect ratios.

The graphical representation of Table 5 is shown in Figure 3 and Figure 4. Figure 3 shows that if the MWCNT volume fraction is increased, the average value of Young’s modulus rises, and a comparison of the base materials shows that a higher percentage of POM in the base hybrid yields a higher Young’s modulus.

Figure 4 shows the Poisson’s ratios results. While the average value of Young’s modulus increases, the average value of the Poisson’s ratio generally decreases, with increases in the MWCNT volume fraction and AR.

The increase in the Poisson’s ratio value in Figure 4 occurs both in the results and in the repeated second runs with a different random seed. In the second run, the value of the Poisson’s ratio obtained for the MWCNT volume fraction of 0.01 changed to 0.34, while the values for the MWCNT volume of fractions 0.005 and 0.016 remained similar, i.e., 0.333 and 0.315, respectively. The third-run values for the MWCNT volume fractions of 0.005, 0.01 and 0.016 were 0.332, 0.308 and 0.324, respectively. The non-monotonic evolution of the Poisson’s ratio with increasing MWCNT volume fraction is likely a numerical outcome of the homogenization procedure used in Ansys Material Designer. At low CNT contents, the shear modulus increases more rapidly than the bulk modulus, leading to a reduction in the Poisson’s ratio. At higher volume fractions, interaction of the reinforcement fields within the representative volume element causes a relatively stronger increase in the bulk modulus, resulting in a slight increase in the Poisson’s ratio. This behaviour reflects the non-linear evolution of the bulk-to-shear modulus ratio rather than experimental dispersion effects.

**Figure 5:**
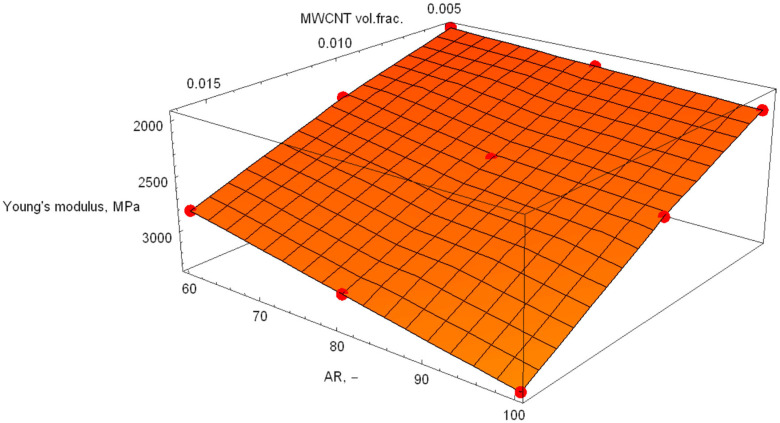
Response surface of the PA6 base material samples.

**Figure 6:**
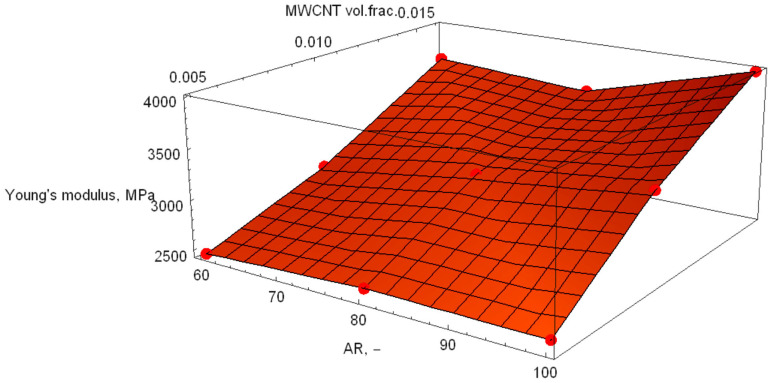
Response surface of the PA6/POM blend base material samples.

**Figure 7:**
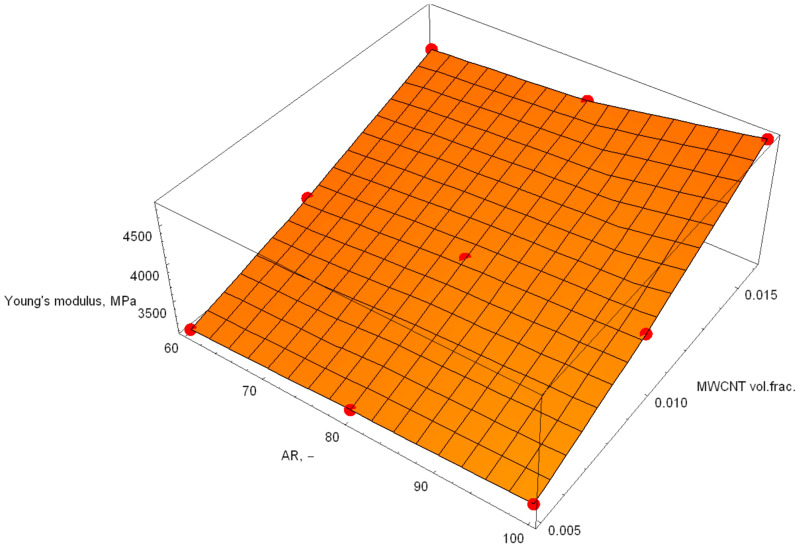
Response surface of the POM blend base material samples.

Figure 5, Figure 6 and Figure 7 show the response surfaces for PA6, blend and POM base materials.

The response surfaces show the predicted Young’s modulus as a function of the MWCNT volume fraction and AR. The slope along the volume fraction axis is steep, indicating that stiffness is primarily governed by reinforcement content. This is expected, since stiffness scales directly with the amount of high-modulus phase. Along the AR direction, the surface rises more gently. Higher AR improves load transfer efficiency (greater effective reinforcement length), but its influence is weaker than that of volume fraction in this range. The absence of strong curvature suggests that there is no pronounced interaction effect between AR and volume fraction, a quasi-linear reinforcement behaviour within the investigated design space and no numerical instability or artificial nonlinearity in the response surface fit.

Using the regression analysis available in Wolfram Mathematica, the following models were calculated:

(12) $$E\left(x,y\right)=848.307+8.44017\cdot x+96181\cdot y$$

(13) $$\left(x,y\right)=1378.07+7.62134\cdot x+108571\cdot y$$

(14) $$E\left(x,y\right)=1955.26+7.91571\cdot x+117170\cdot y$$

where the mathematical model in Formula (12) represents PA6, that in Formula (13) represents the PA6/POM blend, and that in Formula (14) represents POM. In these formulae, x represents the aspect ratio, and y is the MWCN volume fraction. The z-axis responses are the Young’s modulus calculations performed by ANSYS 2019.

This component of the statistical analysis employed only Young’s modulus. During the procedure, the Poisson’s ratio did not exhibit a large change.

Table 6(a)−(c) shows the ANOVA data for the models in Formulas (12)–(14), respectively. The *p*-values are given in Table 6, and the values of $R^{2}$ for the models of that table are shown as follows:

(15) $$R^{2}=0.981$$

(16) $$R^{2}=0.964$$

(17) $$R^{2}=0.986$$

The values of $R^{2}\;$ indicate that over 95% of the total variability in the response data is explained by the differences between groups and under 5% is due to random error.

This final part of the statistical analysis, the ANOVA, indicates that the fitted models are significant. The statistical models and graphs described above show that, within the tested range of the MWCNT volume fractions, the greater the amount of MWCNTs used, the better the mechanical properties. However, rheological behaviour could be a limiting factor. It was earlier shown [19] that high levels of CNTs (above a 5% volume fraction) can create lumps and in turn faults and defects in the material.

This model did not consider the curvature of CNTs. MWCNTs generally feature a low curvature and the MWCNTs here exhibited a mid-range aspect ratio, which means that curvature was very low. Given these limitations, we assume the model to have reasonably accurate results. This study identified tools that aided the choice of additive materials. Certain parameters serve as indicators for the appropriate choice of additives, but the complete mechanical properties of the material samples produced are still required. Results achieved in this study follow the trends found in the work [53,69,70], where the Young’s modulus increased at least 30% with the addition of up to 5 wt% of carbon nanotubes.

## 4. Conclusions

This study explored the impact of the size and volume fraction of MWCNTs on the properties of PA6, POM and their hybrids, using a multi-criterion decision matrix for filler selection, the rule of mixtures and finite-element micromechanical simulations to numerically analyse the elastic modulus of RVEs. This work expands existing knowledge by quantifying the mechanical properties of PA6/POM composites as a function of filler type, the aspect ratio (size) and the volume fraction; these have not been systematically analysed in the literature.

The results can serve as a starting point for designing a new hybrid POM/PA6 with improved mechanical properties while maintaining the positive features of both materials. Adding MWCNTs has a positive impact on Young’s modulus, while it causes a decrease in Poisson’s ratio. At higher aspect ratios (longer nanotubes), Young’s modulus increased. Response surfaces demonstrate that Young’s modulus is predominantly controlled by MWCNT volume fraction, while the aspect ratio provides a secondary stiffening contribution, with an approximately linear and weakly coupled interaction within the investigated parameter space. A possible linear dependence can be used to predict the properties of polymer hybrid materials with added MWCNTs. The mathematical models of the response surfaces of the samples with the PA6/POM blend base material should be further investigated, and any non-linear components should be verified.

The results show that with an increase in aspect ratio from 60 to 100, Young’s modulus increases by 3% to 13%, with the largest improvement observed for the PA6 base material. When examining the influence of the MWCNT volume fraction, an increase from 0.5% to 1.6% results in a predicted average increase of 46% in Young’s modulus, while the Poisson’s ratio decreases by up to 5%. The results show that although the aspect ratio has a clear effect, the effect of MWCNTs on Young’s modulus primarily depends on volume fraction.

The numerical results offer initial guidance when choosing materials for polymer gears and other load-bearing components, but also highlight the limitations of the chopped-fibre RVE approximation, particularly the inability to capture nanotube curvature. We studied MWCNTS, which are generally less curved than SWCNTs and with a mid-range aspect ratio; their curvature is predicted to be very low. This renders our model reliable. The geometrical model designed using the chopped fibre option has only rectilinear fibres. Thus, model reliability should be verified in physical experiments, but the results are consistent with previous research reported in the literature. Since this research revealed a linear trend, a physical experiment with a reduced number of design points is planned.

The rule of mixtures only approximately calculates properties of PA6/POM. Experimental results would provide more precise input data for numerical analysis. Thus, future work will target physical testing, exploration of higher—but still processable—MWCNT loadings and inclusion of economic and manufacturing constraints to support practical material optimisation. Overall, this study establishes a solid foundation for the tailoring hybrid PA6/POM composites via controlled nano-reinforcement, enabling more predictable and efficient design of advanced polymer components.

## Figures and Tables

**Table 1:** Decision matrix.

Property	Material/Weight	Graphite	Nano-Clay	CNTs	Graphene Nanoplatelets	Graphene Oxide
Mechanical properties	0.2	3	3	4	4	4
Thermal stability	0.05	4	4	4	5	4
Flammability	0.05	4	4	4	4	4
Workability	0.1	4	2	2	2	3
Price	0.1	4	4	4	2	2
Local sourcing	0.1	4	4	4	2	2
Sustainability	0.2	3	4	4	2	2
Know-how	0.05	4	4	5	5	5
Research novelty	0.15	3	3	3	5	5
Rating	1	3.45	3.45	3.7	3.25	3.3

**Table 2:** Material properties for input into FEA software (ANSYS 2019 R1).

Material	PA6	POM	POM/PA Blend (Calculated)	MWCNTs
Density, g/cm^3^	1.140	1.410	1.260	2.000
Young’s modulus, GPa	1.600	2.660	2.074	1000
Poisson’s ratio	0.35	0.39	0.368	0.27

**Table 3:** Sample annotations and compositions.

Sample Designation	POM wt%	MWCNTs *d* = 50 nm AR. = 60 Volume%	MWCNTs *d* = 50 nm AR. = 80 Volume%	MWCNTs *d* = 50 nm AR. = 100 Volume%
POM/05NT/AR60	100.0%	0.5%	0%	0%
POM/10NT/AR60	100.0%	1.0%	0%	0%
POM/16N/AR60	100.0%	1.6%	0%	0%
blend/05NT/AR60	50.0%	0.5%	0%	0%
blend/10NT/AR60	50.0%	1.0%	0%	0%
blend/16NT/AR60	50.0%	1.6%	0%	0%
PA6/05NT/AR60	0.0%	0.5%	0%	0%
PA6/10NT/AR60	0.0%	1.0%	0%	0%
PA6/16NT/AR60	0.0%	1.6%	0%	0%
POM/05NT/AR80	100.0%	0%	0.5%	0%
POM/10NT/AR80	100.0%	0%	1.0%	0%
POM/16NT/AR80	100.0%	0%	1.6%	0%
blend/05NT/AR80	50.0%	0%	0.5%	0%
blend/10NT/AR80	50.0%	0%	1.0%	0%
blend/16NT/AR80	50.0%	0%	1.6%	0%
PA6/05NT/AR80	0.0%	0%	0.5%	0%
PA6/10NT/AR80	0.0%	0%	1.0%	0%
PA6/16NT/AR80	0.0%	0%	1.6%	0%
POM/05NT/AR100	100.0%	0%	0%	0.5%
POM/10NT/AR100	100.0%	0%	0%	1.0%
POM/16NT/AR100	100.0%	0%	0%	1.6%
blend/05NT/AR100	50.0%	0%	0%	0.5%
blend/10NT/AR100	50.0%	0%	0%	1.0%
blend/16NT/AR100	50.0%	0%	0%	1.6%
PA6/05NT/AR100	0.0%	0%	0%	0.5%
PA6/10NT/AR100	0.0%	0%	0%	1.0%
PA6/16NT/AR100	0.0%	0%	0%	1.6%

**Table 4:** ANSYS results of one design point with different fibre volume fractions.

Parameters							
Fibre Volume Fraction	0.0051687	3.1341 × 10^−6^	0.010286	6.147 × 10^−6^	0.015908	9.9282 × 10^−6^	
Stiffness Matrix							
D [1,1]	2892	79.637	3213.1	204.42	3988.7	643.12	MPa
D [2,1]	1501.6	23,867	1621.3	85.752	1788.6	119.3	MPa
D [3,1]	1512.8	17,321	1628.6	57.503	1744.7	140.59	MPa
D [4,1]	7.7168	33.499	−28.376	151.65	114.85	195.73	MPa
D [5,1]	16.709	17.098	−13.899	38.953	−18.999	80.078	MPa
D [6,1]	24.601	48.758	24.723	147.91	−72.949	187.19	MPa
D [2,2]	3004.5	59.598	3377.5	235.39	3977.7	409.15	MPa
D [3,2]	1506.3	23.898	1676.3	78.992	1757.7	159.63	MPa
D [4,2]	10.665	47.718	−19.834	148	95.124	209.82	MPa
D [5,2]	23.126	45.937	−92.14	146.66	−103.71	181.31	MPa
D [6,2]	3.7436	19.474	15.398	44.539	9.653	88.983	MPa
D [3,3]	2974.7	121.34	3489.8	261.7	3781.1	352.35	MPa
D [4,3]	10.001	23.321	11.606	41.085	17.722	65.042	MPa
D [5,3]	43.252	29.835	−110.14	139.47	−84.573	139.6	MPa
D [6,3]	35.532	49.658	17.147	106.78	−89.809	164.96	MPa
D [4,4]	709.15	25.117	835.13	87.031	990.17	118.33	MPa
D [5,4]	3.0886	18.946	17.455	47.376	9.9566	89.333	MPa
D [6,4]	16.533	18.446	−15.422	36.99	−17.157	76.393	MPa
D [5,5]	714.7	20.48	884.65	72.873	965.73	157.71	MPa
D [6,5]	11.909	24.017	10.597	41.105	14.817	68.766	MPa
D [6,6]	720.36	17.156	834.55	60.375	955.15	142.89	MPa

**Table 5:** Summarised results.

	Young’s Modulus, MPa			Poisson’s Ratio			Base Material
MWCNTs Volume Fraction	0.005	0.01	0.016	0.005	0.01	0.016	
AR							
60	1938.4	2276.4	2809.3	0.334	0.319	0.316	PA6
80	2024.1	2493	3078.4	0.332	0.308	0.324	PA6
100	2102.9	2591	3343	0.33	0.316	0.312	PA6
60	2454.2	2871	3621.4	0.353	0.336	0.32	Blend
80	2540.3	3127.4	3511.5	0.346	0.33	0.329	Blend
100	2539.3	3328.5	3993.4	0.357	0.351	0.339	Blend
60	3103.8	3577.1	4268.9	0.376	0.36	0.361	POM
80	3196.7	3756.4	4391.1	0.376	0.356	0.362	POM
100	3268.4	3863.3	4767.9	0.373	0.366	0.356	POM

**Table 6:** ANOVA results.

$ANOVA\to \begin{array}{cccccc} “” & “DF” & “SumOfSq” & “MeanSq” & “FRatio” & “pValue”\\ x & 2 & 171905.26688740053 & 85952.63344370027 & 9.57766727048538 & 0.029841308525907037\\ y & 2 & 1688212.529840671 & 844106.2649203358 & 94.05842057922084 & 0.00043350000637573937\\ “Error” & 4 & 35897.10563805975 & 8974.276409514938 & “” & “”\\ “Total” & 8 & 1896014.902366132 & “” & “” & “” \end{array},CellMeans\to \begin{array}{cc} “All” & 2517.3903601003085\\ x[60] & 2341.3693530294277\\ x[80] & 2531.8255171066194\\ x[100] & 2678.976210164878\\ y[0.005] & 2021.7858268470475\\ y[0.01] & 2453.5022917186843\\ y[0.016] & 3076.8829617351935 \end{array}$	(a)
$ANOVA\to \begin{array}{cccccc} “” & “DF” & “SumOfSq” & “MeanSq” & “FRatio” & “pValue”\\ x & 2 & 150620.51605990902 & 75310.25802995451 & 3.463112953842229 & 0.13402309145416913\\ y & 2 & 2151021.28118594 & 1075510.64059297 & 49.45693891994381 & 0.0015106786685598738\\ “Error” & 4 & 86985.6213571086 & 21746.40533927715 & “” & “”\\ “Total” & 8 & 2388627.418602957 & “” & “” & “” \end{array},CellMeans\to \begin{array}{cc} “All” & 3109.671886963193\\ x[60] & 2982.2081801780623\\ x[80] & 3059.7456041192186\\ x[100] & 3287.061876592299\\ y[0.005] & 2511.269430214536\\ y[0.01] & 3108.974096195742\\ y[0.016] & 3708.772134479302 \end{array}$	(b)
$ANOVA\to \begin{array}{cccccc} “” & “DF” & “SumOfSq” & “MeanSq” & “FRatio” & “pValue”\\ x & 2 & 151821.57966545178 & 75910.78983272589 & 7.793915137619163 & 0.04170108147329964\\ y & 2 & 2502180.757687063 & 1251090.378843531 & 128.45199297339664 & 0.0002350490793864818\\ “Error” & 4 & 38959.00249995005 & 9739.750624987513 & “” & “”\\ “Total” & 8 & 2692961.339852465 & “” & “” & “” \end{array},CellMeans\to \begin{array}{cc} “All” & 3799.277483048905\\ x[60] & 3649.9116325632313\\ x[80] & 3781.380828240702\\ x[100] & 3966.5399883427835\\ y[0.005] & 3189.6110141434824\\ y[0.01] & 3732.277120578919\\ y[0.016] & 4475.944314424316 \end{array}$	(c)

## Data Availability

Results of numerical simulation are available under 10.6084/m9.figshare.31009318.

## Abbreviations

The following abbreviations are used in this manuscript:

PA6	polyamide 6
POM	polyoxymethylene
MWCNTs	multi-walled carbon nanotubes
RVE	representative volume element
PA	polyamide
CNTs	carbon nanotubes
CF	carbon fibre
FEM	finite element method
FEA	finite element analysis
SWCNTs	single-walled carbon nanotubes
